# The JDRF CCTN CGM TIME Trial: Timing of Initiation of continuous glucose Monitoring in Established pediatric type 1 diabetes: study protocol, recruitment and baseline characteristics

**DOI:** 10.1186/1471-2431-14-183

**Published:** 2014-07-18

**Authors:** Margaret L Lawson, Brenda Bradley, Karen McAssey, Cheril Clarson, Susan E Kirsch, Farid H Mahmud, Jacqueline R Curtis, Christine Richardson, Jennilea Courtney, Tammy Cooper, Cynthia J Downie, Gopalan Rajamannar, Nicholas Barrowman

**Affiliations:** 1 Division of Endocrinology and Metabolism, Children’s Hospital of Eastern Ontario, University of Ottawa, Ottawa, ON, Canada; 2 CHEO Research Institute, Ottawa, ON, Canada; 3 McMaster Children’s Hospital, Hamilton, ON, Canada; 4 Children’s Hospital, London Health Sciences Centre, London, ON, Canada; 5 Markham-Stouffville Hospital, Markham, ON, Canada; 6 Hospital for Sick Children, Toronto, ON, Canada; 7 Children’s Hospital of Eastern Ontario, Ottawa, ON, Canada; 8 Robarts Clinical Trials Inc, London, ON, Canada

**Keywords:** Continuous glucose monitoring, Continuous subcutaneous insulin infusion, Type 1 diabetes, Adherence, Pediatrics, Glycosylated hemoglobin, Children, Adolescents, Quality of life, Treatment satisfaction

## Abstract

**Background:**

Continuous glucose monitoring (CGM) has been shown to improve glucose control in adults with type 1 diabetes. Effectiveness of CGM is directly linked with CGM adherence, which can be challenging to maintain in children and adolescents. We hypothesize that initiating CGM at the same time as starting insulin pump therapy in pump naïve children and adolescents with type 1 diabetes will result in greater CGM adherence and effectiveness compared to delaying CGM introduction by 6 months, and that this is related to greater readiness for making behaviour change at the time of pump initiation.

**Methods/Design:**

The CGM TIME Trial is a multicenter randomized controlled trial. Eligible children and adolescents (5-18 years) with established type 1 diabetes were randomized to simultaneous initiation of pump (Medtronic Veo©) and CGM (Enlite©) or to standard pump therapy with delayed CGM introduction. Primary outcomes are CGM adherence and hemoglobin A1C at 6 and 12 months post pump initiation. Secondary outcomes include glycemic variability, stage of readiness, and other patient-reported outcomes with follow-up to 24 months. 144 (95%) of the 152 eligible patients were enrolled and randomized. Allowing for 10% withdrawals, this will provide 93% power to detect a between group difference in CGM adherence and 86% power to detect a between group difference in hemoglobin A1C. Baseline characteristics were similar between the treatment groups. Analysis of 12 month primary outcomes will begin in September 2014.

**Discussion:**

The CGM TIME Trial is the first study to examine the relationship between timing of CGM initiation, readiness for behaviour change, and subsequent CGM adherence in pump naïve children and adolescents. Its findings will advance our understanding of when and how to initiate CGM in children and adolescents with type 1 diabetes.

**Trial registration:**

ClinicalTrial.gov NCT01295788. Registered 14 February 2011.

## Background

Continuous glucose monitoring (CGM) provides glucose measurements in real-time updated every 5 minutes to an external monitor or insulin pump. CGM has been shown to significantly improve glycemic control in type 1 diabetes (T1D), with the degree of benefit directly related to frequency of CGM use [1-3]. Children and adolescents appear less willing to wear CGM than their adult counterparts, resulting in reduced effectiveness in the pediatric population [3,4].

Most CGM studies have enrolled experienced pump or multiple daily injection (MDI) users, believing that successful initiation of CGM requires experience with the insulin delivery system [2,4-6]. However, these studies and clinical experience suggest that when CGM is added to the regimen of existing pump users, many consider it as an optional tool to be used on an intermittent basis rather than an integral and necessary part of effective pump management. Successful pump users may believe that they are doing well enough without CGM, while those who are struggling with the demands of pump therapy may perceive CGM as being overly burdensome. In contrast, at the time of pump initiation, children and adolescents, and their parents, are highly motivated to make behaviour changes that they believe will improve diabetes control and quality of life. Indeed, several studies have observed that CGM adherence is greater when CGM is initiated at the same time as starting pump therapy [7-11].

We hypothesize that simultaneous initiation of pump and CGM will be more effective than standard pump therapy with initiation of CGM 6 months later, and that this is related to greater readiness for making and sustaining behaviour change at the time of pump initiation. The CGM TIME Trial (**T**iming of **I**nitiation of Continuous Glucose **M**onitoring in **E**stablished Pediatric Diabetes) was designed to test this hypothesis and launched as part of the newly created JDRF Canadian Clinical Trial Network (JDRF CCTN). This paper describes the research design and methods of the CGM TIME Trial, recruitment, and baseline characteristics of the 144 subjects enrolled in the trial.

## Methods/Design

### Protocol development

The initial protocol for the CGM TIME Trial, developed by investigators at the Children’s Hospital of Eastern Ontario (CHEO), was based on a feasibility pilot study conducted at CHEO and St. Justine’s Hospital in Montreal, Quebec [12]. After funding approval from the JDRF CCTN, the protocol was revised based on input from the JDRF CCTN Steering Committee, the CGM TIME Trial Study Group, and international consultation with experts in CGM trials. Four additional pediatric diabetes centers, all located in Southern Ontario, Canada, were selected to participate in the study. Institutional review board approval was obtained from each site’s ethics board (The CHEO Research Ethics Board, the University of Western Ontario Research Ethics Board for Health Sciences Research Involving Human Subjects, the Hamilton Health Sciences/McMaster Health Sciences Research Ethics Board, the Research Ethics Committee of Markham Stouffville Hospital, and the Research Ethics Board for The Hospital for Sick Children).

### Study design

**Figure 1:**
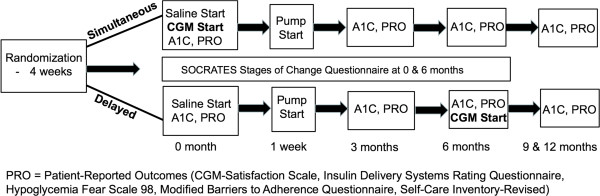
Study design.

The CGM TIME Trial is a 12-month multicenter randomized controlled trial with an optional 6-month Extension Phase. Children and adolescents, 5–18 years of age, with type 1 diabetes duration > 1 year who were initiating insulin pump therapy were randomly assigned to simultaneous initiation of pump therapy and CGM (Simultaneous Group) or to starting standard pump therapy with addition of CGM six months later (Delayed Group) (Figure 1 – Study Design). Randomization was performed centrally, stratified by study center and by age (5–12 years vs. 13–18 years), using a computer-generated randomization schedule with variable block size.

### Study population

Potential participants were required to meet their local site’s criteria for starting insulin pump therapy. Trial eligibility criteria included: age 5–18 years; type 1 diabetes duration of at least 1 year; naïve to pump therapy; ready to start pump therapy with the Medtronic Veo© pump; willing to use CGM and be randomized to simultaneous or delayed CGM initiation; home computer with internet access; and ability of the parent and/or child to speak and read English or French. Subjects were excluded if they had conditions which in the opinion of the investigator would interfere with the subject’s ability to participate in the study; had received oral and/or intravenous steroid therapy on more than 2 occasions in the previous 12 months; had used real-time CGM for more than 50% of the previous 6 months; were currently enrolled in another intervention trial; or had a sibling who had participated in the CGM TIME Trial. There were no upper or lower limits for baseline A1C values. Ethnicity was tabulated by self-report. Informed consent of the parent, plus assent of the child where indicated, was required before enrolment.

### Study intervention

#### Standard therapy for both groups

All subjects received standard diabetes care for children and adolescents starting insulin pump therapy at these sites which included: 1) Participation in a standardized pump education program divided into two sessions approximately one week apart; week one was a saline start (i.e., continued delivery of insulin via injections with saline used in the insulin pump to allow practice with bolusing and set changes) with transition to insulin pump therapy the following week; 2) Training on Medtronic’s CareLink Personal software with instructions to upload the insulin pump daily during the 10 days after pump initiation and weekly thereafter; 3) Daily telephone contact for the purpose of insulin adjustment with their pump nurse or diabetes physician for 10 days following the pump start; 4) Diabetes clinic visits at three, six, nine and twelve months after pump initiation; and 5) Telephone assessment and education session with the pump nurse one month after pump initiation. The content and structure of the telephone sessions was standardized and focused on insulin adjustment and use of the pump for both groups. The Simultaneous Group also received support for CGM during these calls. Comparable support was provided to all subjects in the Delayed Group when CGM was started at 6 months, with telephone calls on four separate days within the 10 day period after the CGM start, and one month later. Simultaneous subjects received the same telephone contact after the six month visit. Following the 12-month study visit, subjects participating in the optional Extension Phase returned to routine clinical follow-up with their diabetes team with follow-up visits at 15 and 18 months.

#### Pump and CGM devices

At the time of recruitment, Medtronic had the only CGM device licensed for use in Canada. All subjects used the Medtronic Veo© pump and were provided with a Contour© BG meter that transmits BG values via radio frequency to the Veo© pump for use with the Bolus Wizard, CGM calibration and data storage. Pump training used standardized written teaching materials for the Medtronic Veo© pump. Both Simultaneous and Delayed Group subjects used the Medtronic Enlite© sensor (under a Health Canada Investigational Testing Authorization until fully approved by Health Canada), Minilink transmitter, and CareLink Personal software. CGM education was standardized using written teaching materials, with the only difference between the groups being the timing of CGM teaching and initiation (i.e., during the pump start or 6 months later). Training and programming of pump and CGM settings were standardized for all subjects (Additional file 1: Figure S1 and Additional file 2: Figure S2).

#### Simultaneous group

Subjects randomized to the Simultaneous Group were started on CGM during week one of the pump training (the saline start). During this week, they were instructed to make observations about sensor glucose readings relative to BG levels but not to use the CGM data to adjust insulin therapy. Families were instructed not to activate Low Glucose Suspend (LGS) during this week. When insulin pump therapy was initiated the following week, LGS was activated and other CGM settings initiated in a standardized step-wise fashion (Additional file 2: Figure S2). During the daily telephone contact, families received reinforcement on the interpretation and use of CGM and Self-Monitoring of Blood Glucose (SMBG) for insulin therapy adjustments.

#### Delayed group

Subjects randomized to the Delayed Group started standard pump therapy as described above. Pump setting adjustments were based on SMBG from pump initiation until the 6-month visit at which they received the standardized training on CGM and were instructed on the interpretation and use of CGM and SMBG to adjust insulin therapy, with reinforcement through telephone calls during the next 10 days and one month later. Delayed Group subjects were instructed to upload pump and CGM data daily to CareLink Personal during the 10 days after CGM initiation and weekly thereafter. Pump and CGM settings were standardized as with the Simultaneous Group (Additional file 1: Figure S1 and Additional file 2: Figure S2).

### Outcome assessments

Outcome assessments include CGM adherence, A1C, CGM glucose profiles, and patient-reported outcomes (Additional file 3: Figure S3). The *primary outcome* is CGM adherence (number of hours of CGM use per week) obtained from CareLink Professional which accessed data from the families’ weekly upload to CareLink Personal. The *main secondary outcome* is A1C, evaluated by centralized A1C measurement at baseline, 6 and 12 months (Roche Diagnostics Turbidimetric Inhibition Immunoassay, utilizing the DCCT/NGSP formula, Dynacare Laboratories, Toronto, Canada). Additional local A1Cs are obtained at baseline and every study visit throughout the trial (DCA2000, Bayer Diagnostics, Tarrytown, NY or local lab BioRad Variant II by HPLC).

CGM profiles are obtained on all subjects at baseline, six and twelve months. At baseline, Delayed Group subjects wear the iPro2 for the 6 days prior to initiation of pump therapy while Simultaneous Group subjects wear CGM during this same time period (the saline start) but are instructed not to use the CGM data to adjust insulin therapy during this week. The six month CGM profile for Delayed Group subjects are based on a 6 day iPro2 recording completed during the week prior to the study visit, while for the Simultaneous Group it is based on CareLink data if subjects are wearing CGM for at least 6 days prior to the six month study visit or if not, a 6 day iPro2 recording is completed prior to the study visit. Similarly, at 12 months, CGM profiles for both groups are based on CareLink data if wearing CGM at least 6 days prior to the study visit or a 6 day iPro2 recording.

Subjects (if > 10 years of age) and their parents complete questionnaires at each study visit including: *Stages of Change Readiness and Treatment Eagerness Scale (SOCRATES – Diabetes versio*n) [13], *Self-Care Inventory – Revised (SCI-R)*[14], *Modified Barriers to Adherence Questionnaire (MBAQ)*[15], *Insulin Delivery Systems Rating Questionnaire (IDSRQ)*[16], *CGM Satisfaction Scale (CGM-SAT)*[17], *Low Blood Sugar Survey* (also known as the *Hypoglycemia Fear Scale (HFS-98*) [18]*,* and a questionnaire developed for this study on supplemental health insurance and socioeconomic status.

### Sample size estimation

Sample size calculation was based on the primary outcome measure: CGM use (in hours per week) six months after CGM initiation (i.e., the 4 week time period before the six month visit for the Simultaneous Group and before the twelve month visit for the Delayed Group). A clinically meaningful difference in CGM use is 30 hours per week, which for example, would translate into an increase in CGM use from 49% to 69% of the time. Assuming a standard deviation (SD) of 56.4 hours (weighted average of SDs in the 15–24 and 8–14 year age groups in two pediatric studies [2,8]) and allowing for a 10% drop-out rate, 64 subjects per group are required with a type 1 error rate at 0.05 and power of 80%.

The study was also powered to detect a difference between the groups in the change in A1C from baseline to 6 or 12 months. Assuming a clinically meaningful difference in A1C of 0.5% and a standard deviation of 0.93 (weighted average of SDs at 6 months from two pediatric CGM studies [2,8]), a two-sample *t* test with a power of 80% and a type 1 error rate of 0.05 will require 63 subjects per group allowing for a 10% drop-out rate. To increase the power for other secondary outcomes including the patient-reported outcomes, sample size was set at a minimum of 128 and maximum of 150 subjects.

### Statistical analyses

Efficacy analyses will be performed according to the intent-to-treat (ITT) principle and will include all randomized subjects who complete at least one study visit after randomization. Extension Phase analyses will include all subjects who enroll in the Extension Phase and complete at least one study visit after the twelve month visit. Statistical tests will be two-sided and performed at the 0.05 level of significance.

The primary efficacy analysis, CGM adherence, will be evaluated by comparing CGM adherence (hours per week) computed over the four weeks prior to the six month visit for the Simultaneous Group, and over the four weeks prior to the twelve month visit for the Delayed Group using analysis of covariance, with gender, investigative site, and baseline A1C, age, diabetes duration, and body mass index, as covariates. A similar approach will be used for the secondary efficacy parameter of change in A1C from baseline. This analysis will compare the change in A1C between baseline and six months (Simultaneous CGM and pump initiation versus standard pump therapy) and between baseline and twelve months (Simultaneous CGM and pump initiation versus standard pump therapy with delayed CGM initiation).

The association between SOCRATES baseline readiness for change score and CGM adherence computed over the six month period between pump initiation and the six month visit for the Simultaneous Group, and for the Delayed Group over the six month period between the six and the twelve month visits will be assessed using multiple linear regression. Similarly, change in A1C from baseline to 6 months after pump initiation and CGM adherence over the 6 months after its initiation will be assessed using multiple linear regression. These regression models will include gender, investigative site, baseline A1C, age group, diabetes duration and body mass index as predictor variables. Treatment effects on other continuous secondary outcomes will be assessed using analysis of covariance, adjusting for gender, investigative site, baseline A1C, age group, diabetes duration and body mass index.

Logistic regression, with gender, investigative site and age group as strata will be used to examine the differences between study groups with regards to the proportion of subjects achieving specific target A1C values (percentage of subjects with A1C level of < 7.0%, ≤ 7.5%, and ≤ 8.0%) at 6 and 12 months with calculations of odds ratios and their 95% CI. Similar analyses will be performed to examine the differences between study groups with regards to an absolute change in A1C ≥ or ≤ 0.5% at 6 and 12 months.

### Study recruitment and baseline characteristics

**Figure 2:**
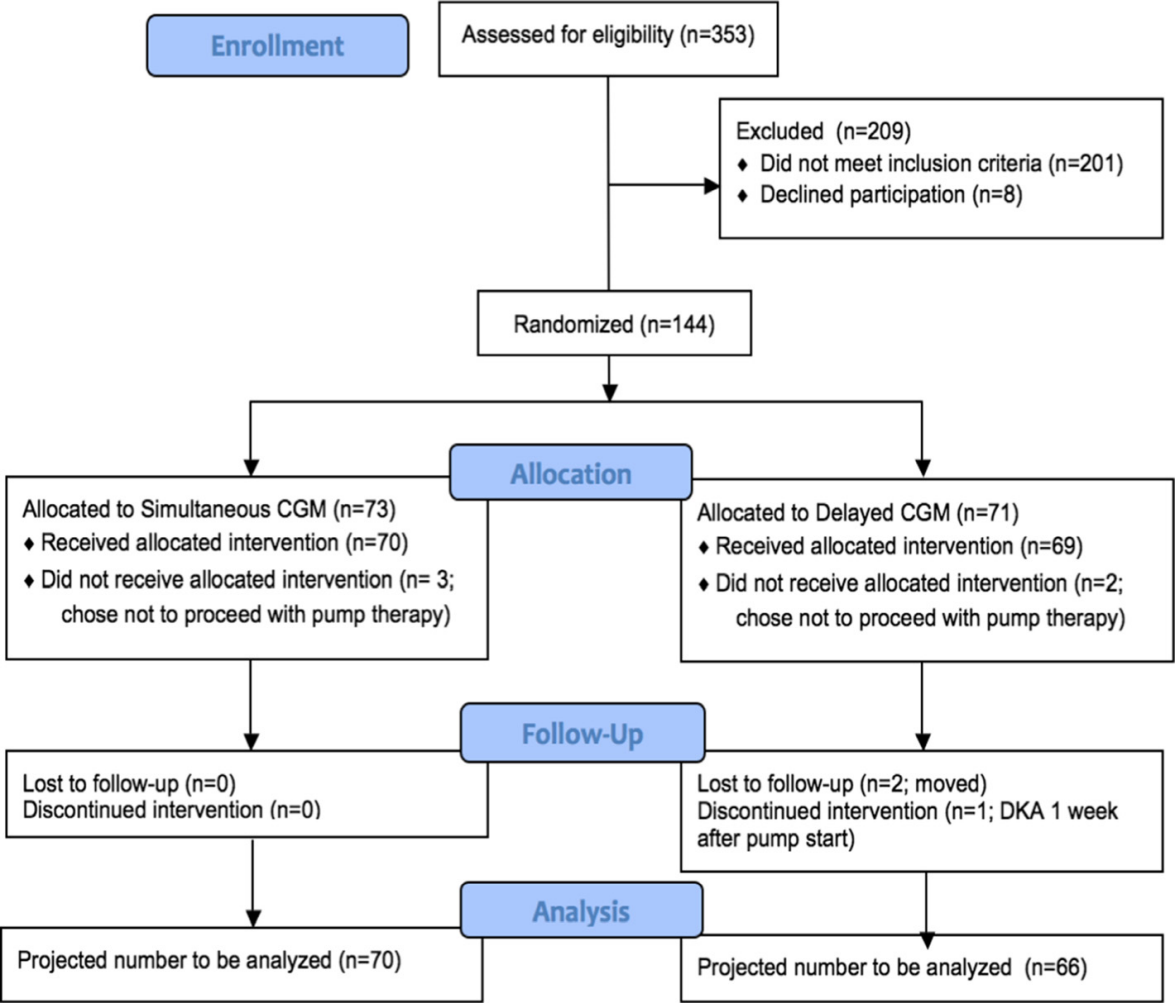
CONSORT flow diagram.

Recruitment began at the lead site (CHEO) on June 30, 2011 with all 5 sites operational by October 15, 2011. The minimum sample size of 128 was reached on March 19, 2013. Recruitment closed on May 31, 2013 with 144 randomized subjects (Figure 2 – Consort Flow Diagram). Allowing for 10% withdrawals, this will provide 93% power to detect a between group difference at six and twelve months for CGM adherence and 86% power to detect a between group difference in A1C.

Clinical statistics at the 5 sites for the previous two years showed an average 240 pump starts per year (4 per center per month). Based on the experience of the pilot study, we projected that 50% of candidates for pump start would meet the trial eligibility criteria and that 50% of these would consent to study participation, thus requiring 26 months to reach the minimum sample size of 128. Actual pump starts were lower than predicated at 2.8 per center per month with 43.0% of pump start candidates meeting all eligibility criteria. However, 94.7% of the 152 eligible pump start candidates consented to participation and entered the study. This resulted in the minimum sample size of 128 being reached after 19 months. Recruitment was extended an additional 2 months to increase power for secondary outcome measures and protect against early terminations.

The 201 patients who were not eligible for the study were excluded because they: chose a non-Medtronic pump [but met all other inclusion criteria] (53.2%), were not interested in CGM (20.8%), were < 5 years of age (9.0%), had < 1 year T1D duration (7.0%), were deemed ineligible by the investigator (3.5%), were not naive to pump therapy (2.0%), were not willing to be randomized to delayed CGM (1.5%), or lacked home internet access (0.5%). Five subjects (2.5%) were excluded for “other” reasons which included 2 subjects with an uncertain T1D diagnosis, 2 from out of province, and 1 > 18 years of age. Of the 7 subjects excluded due to investigator’s opinion, 3 had developmental delay and 4 had parental issues which were considered to interfere with trial participation.

There were no significant differences in baseline characteristics between the Simultaneous and Delayed Groups (Table 1). There were 8 early terminations (5.6% of 144) prior to the main study’s 12 month visit. Five subjects terminated because they decided not to start pump therapy (3 Simultaneous and 2 Delayed Group subjects), 2 Delayed Group subjects withdrew from the study after the 6 month visit, having decided not to start CGM, because they planned to receive diabetes care at another center and were not willing to return for study follow-up, and 1 Delayed Group subject withdrew from the study after the attending physician removed the child from pump therapy one week after pump initiation because pump mismanagement resulted in diabetic ketoacidosis.

**Table 1:** Baseline demographics of recruited participants

	**Simultaneous group (n = 73)**	**Delayed group (n = 71)**	**All subjects (n = 144)**	**p-value (between groups)**
Mean age +/− SD [years (range)]	12.0 +/− 3.3 [5.1-18.1]	12.0 +/− 3.4 [5.1-17.8]	12.0 +/− 3.3 [5.1-18.1]	0.95
Age 5–12 years [n (%)]	39 (53.4%)	33 (46.5%)	72 (50.0%)	0.40
Age 13–18 years [n (%)]	34 (48.6%)	38 (53.5%)	72 (50.0%)	
Gender [n (%)]				
Female	31 (42.5%	32 (45.1%)	63 (43.8%)	0.75
Male	42 (57.5%)	39 (54.9%)	81 (56.3%)	
Mean baseline A1C +/− SD [% (range)]	(n = 71)	(n = 68)	(n = 139)*	0.40
	8.06 +/− 0.99 [5.7-11.2]	7.92 +/− 0.94 [5.5-9.8]	7.99 +/− 0.96 [5.5-11.2]	
Mean diabetes duration +/− SD [years (range)]	(n = 72)	(n = 69)	(n = 141)**	0.58
	3.5 +/− 3.1 [1-14]	3.2 +/− 2.9 [1-16]	3.3 +/− 3.0 [1-16]	
Race or ethnicity [n (%)]				
Caucasian	64 (87.7%)	61 (85.9%)	125 (86.8%)	0.27
African-American	3 (4.1%)	0 (0.0%)	3 (2.1%)	
Asian	2 (2.7%)	4 (5.6%)	6 (4.2%)	
Hispanic	0 (0.0%)	0 (0.0%)	0 (0.0%)	
Other	4 (5.5%)	6 (8.5%)	10 (6.9%)	

*A1C not collected on 3 subjects who withdrew before visit 1; baseline A1C not available for 2 subjects.

**Diabetes duration not available for 3 subjects who withdrew before visit 1.

### Trial organization

The trial is supported, financially and organizationally, by the JDRF CCTN, a new initiative launched in 2010 by a joint partnership between JDRF Canada and the Federal Government of Canada. The lead site of the CGM TIME Trial, the Children’s Hospital of Eastern Ontario, is a JDRF CCTN Clinical Center which provides the infrastructure for the trial’s operations. The study group is comprised of pediatric endocrinologists, diabetes nurse educators, diabetes dietitians, and research coordinators in each of the 5 clinical sites. Robarts Clinical Trials located in London, Ontario, provides study and data management and coordinates reporting for clinical and device-related adverse events. Review of adverse events and data safety monitoring is provided by an independent Data Safety Monitoring Board comprised of experts in the fields of pediatric diabetes, pump therapy and CGM, and clinical trials.

## Discussion

The CGM TIME Trial is the first study to examine the relationship between timing of CGM initiation, readiness for behaviour change, and subsequent CGM adherence in children and adolescents. It was designed to address two previously unanswered questions. First, is simultaneous initiation of CGM and insulin pump therapy more effective, in terms of sustained CGM adherence and A1C reduction, than starting CGM six months after initiation of standard pump therapy? Second, does simultaneous initiation of CGM and insulin pump therapy result in better glycemic control than standard pump therapy with SMBG?

The CGM TIME Trial incorporates several key features which distinguish it from other pediatric CGM trials. First, unlike other pediatric CGM trials [2,4,5,7,8], the CGM TIME Trial did not use a run-in period to pre-select or screen subjects likely to adhere to CGM, and thus offers broader external validity and generalizability to the real-life application of CGM in the pediatric population. Indeed, 94.5% of eligible patients starting pump therapy in the participating sites during the recruitment period consented to trial participation. Second, all subjects are using Medtronic’s Enlite© sensor, the Veo© pump which incorporates Low Glucose Suspend, and the iPro2, making the TIME Trial one of the first pediatric trials of these devices and, at12 months duration, the longest to date. Previous pediatric trials of these devices have ranged from 16 days to 6 months [19-21] but did not examine adherence or effect on A1C. Third, the TIME Trial developed an innovative and standardized approach to patient education and CGM and pump settings. This focused on stepwise integration of alarms to minimize alarm fatigue and annoyance, and the use of study-specific algorithms for responding to CGM trend arrows. It has been suggested that previous CGM trials may have failed to demonstrate effectiveness because they lacked standardization of patient education and treatment algorithms [22]. Fourth, the TIME Trial incorporates multiple patient-reported outcomes including two not previously included in pediatric CGM trials, the SOCRATES questionnaire to measure readiness for change and the Modified Barriers to Adherence Questionnaire.

Readiness for making and sustaining behaviour change, a measure of patient activation, has been linked with subsequent metabolic control in adults with diabetes [23], with subsequent behaviour in a clinical trial amongst adolescents with type 1 diabetes [24], and with adolescents’ readiness to make changes in the balance of responsibility for diabetes management with their parents [25]. Parental readiness for change related to their children’s health has been studied in other chronic conditions such as obesity and polycystic ovarian syndrome, and shown to positively impact outcome [26,27]. There is also evidence linking adolescents’ readiness for behaviour change related to substance abuse [28]. The TIME Trial is the first study to examine the hypothesis that readiness for change in children and youth with type 1 diabetes, and their parents, will predict future CGM adherence and effectiveness, and that readiness for change will be greater at the time of pump initiation compared to six months later.

The TIME Trial has not faced the recruitment and retention challenges commonly faced by clinical trials [29]. We had predicted that it would take 26 months to recruit the planned sample size of 128 but we were able to exceed this, randomizing 144 subjects in 21 months. We attribute this success to multiple factors including the use of the Enlite© sensor which was not available in routine care during most of the recruitment period, the highly engaged and motivated study group members who were participating in the first multicenter trial launched in the JDRF CCTN, and the use of a research recruitment tool modeled after patient decision aids [30].

The CGM TIME Trial has successfully completed enrolment to the first multicenter trial comparing simultaneous pump and CGM initiation to starting standard pump therapy with later addition of CGM. The results of the TIME Trial will advance our understanding of how to initiate CGM and maximize its effectiveness in the pediatric population.

## Abbreviations

A1C: Hemoglobin A1C; BG: Blood glucose; CGM: Continuous glucose monitoring; CGM-SAT: CGM Satisfaction Scale; CHEO: Children’s Hospital of Eastern Ontario; IDSRQ: Insulin Delivery Systems Rating Questionnaire; HFS-98: Hypoglycemia Fear Scale; JDRF CCTN: JDRF Canadian Clinical Trial Network; MBAQ: Modified Barriers to Adherence Questionnaire; MDI: Multiple daily injections; SCI-R: Self-Care Inventory – Revised; SMBG: Self-monitoring of blood glucose; SOCRATES: Stages of Change Readiness and Treatment Eagerness Scale; T1D: Type 1 diabetes.

## Competing interests

This is an investigator-initiated trial. Pumps and CGM supplies were purchased by the Study Group from Medtronic Canada at a discounted price. MLL has been a speaker, without honorarium, at educational events sponsored by Medtronic and Animas with travel reimbursement to attend these events. CC has been a speaker with honorarium at educational events sponsored by Medtronic. KM and SEK have been speakers with honorarium at educational events sponsored by Medtronic and Animas. The other authors have no competing interests to disclose.

## Authors’ contributions

MLL conceived and designed the design, led its coordination, participated in data acquisition, and drafted the initial manuscript; BB, KM, CC, SEK, FHM, JRC, CR, JC, TC, CJD participated in the design of the study, acquisition of data, and provided critical revision of the manuscript; GR and NB developed the statistical analysis plan and provided critical revision of the manuscript; all other listed authors and members of the CGM TIME Trial Study Group participated in the acquisition of data, and provided critical review and final approval of the submitted manuscript. All authors read and approved the final manuscript.

## Authors’ information

The CGM TIME Trial Study Group Members:

Personnel are listed as (PI) for Principal Investigator, (I) for Co-investigator, (C) for Co-ordinators, (DNE) for Diabetes Nurse Educators, (RD) for Dietitians. Children’s Hospital of Eastern Ontario: Margaret L. Lawson (PI), Brenda Bradley (Project Manager), Christine Richardson (DNE), Jennilea Courtney (C), Tammy Cooper (RD). McMaster Children’s Hospital: Karen McAssey (I), Janice Muileboom (DNE), Anne Marie DiGravio (RD), Elizabeth Helden (C), Amiee Hill (C). Children’s Hospital, London Health Sciences Centre: Cheril Clarson (I), Chantelle Black (C), Ruth Duncan (C), Keira Evans (DNE), Jenna MacIsaac (RD), Margaret Watson (C). Markham-Stouffville Hospital: Susan E. Kirsch (I), Alanna Landry (DNE), Marilyn Fry (RD), Sameer Datwani (C). Hospital for Sick Children: Farid H Mahmud (I), Jacqueline R Curtis (I), Lynne Cormack (DNE), Kamaljeet Sahota (C), Vanita Pais (RD). Coordinating Center: Robarts Clinical Trials: Cynthia J Downie, Liz Liddiard, Dildeep Kaur, Melody Chow, Helen Sun. Biostatisticans: Gopalan Rajamannar PhD, Robarts Clinical Trials; Nicholas Barrowman PhD, CHEO Research Institute. JDRF Canadian Clinical Trial Network: Olivia Lou, Concepcion Nierras. Data Safety Monitoring Board: Heather J. Dean (Chair), William V. Tamborlane, Howard A. Wolpert.

## Pre-publication history

The pre-publication history for this paper can be accessed here:

http://www.biomedcentral.com/1471-2431/14/183/prepub

## Supplementary Material

Additional file 1: Figure S1 Standardized Settings for Pump and CGM Initiation. Click here for file

Additional file 2: Figure S2 Stepwise Integration of CGM Alarms. ISF = insulin sensitivity factor. ^1^ Delayed Group: all dates are following initiation of CGM at the 6 month visit. ^2^ Decision to set alarms is based on assessment of BG variability within and between days; goal is to have < 1 alarm per day. ^3^Study-specific guidelines developed for the CGM TIME Trial. Click here for file

Additional file 3: Figure S3 Schedule of Study Visits, Telephone Contacts, and Outcome Assessments. CareLink uploads performed weekly for the duration of the trial. 24 month followup involves data from CareLink uploads and local A1Cs at 18 and 24 months. Questionnaires: SOCRATES = Stages of Change Readiness and Treatment Eagerness Scale, SCI-R = Self-Care Inventory-Revised, MBAQ = Modified Barriers to Adherence, IDRSQ = Insulin Delivery Systems Rating Questionnaire, CGM-SAT = CGM Satisfaction Scale, HFS-98 = Hypoglycemia Fear Scale, SES = Health Insurance and Socioeconomic Status Questionnaire. Click here for file
