# Supplementary figures and images for: The JDRF CCTN CGM TIME Trial: Timing of Initiation of continuous glucose Monitoring in Established pediatric type 1 diabetes: study protocol, recruitment and baseline characteristics
Source: BMC Pediatr. 2014 Jul 18;14:183. doi: 10.1186/1471-2431-14-183 (PMC4109785; doi:10.1186/1471-2431-14-183)

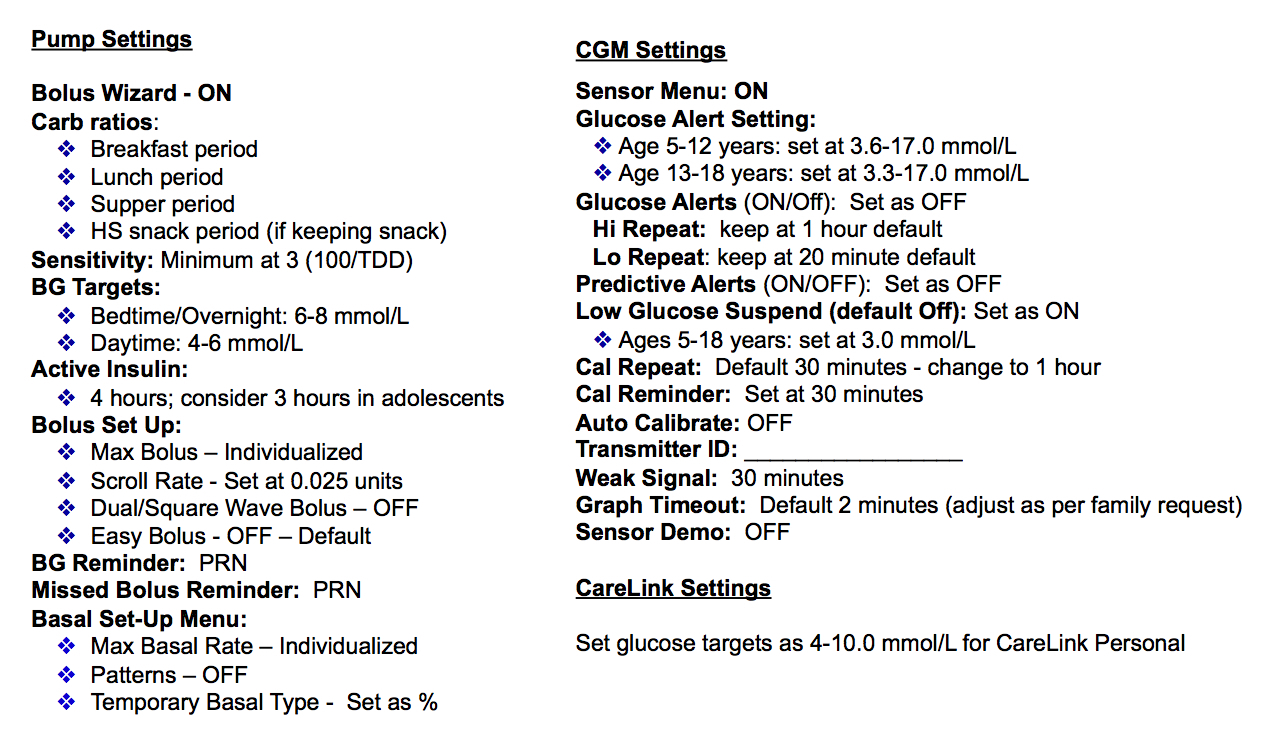

Supplement: Additional file 1: Figure S1 — Standardized Settings for Pump and CGM Initiation. [file 1471-2431-14-183-S1.jpeg]

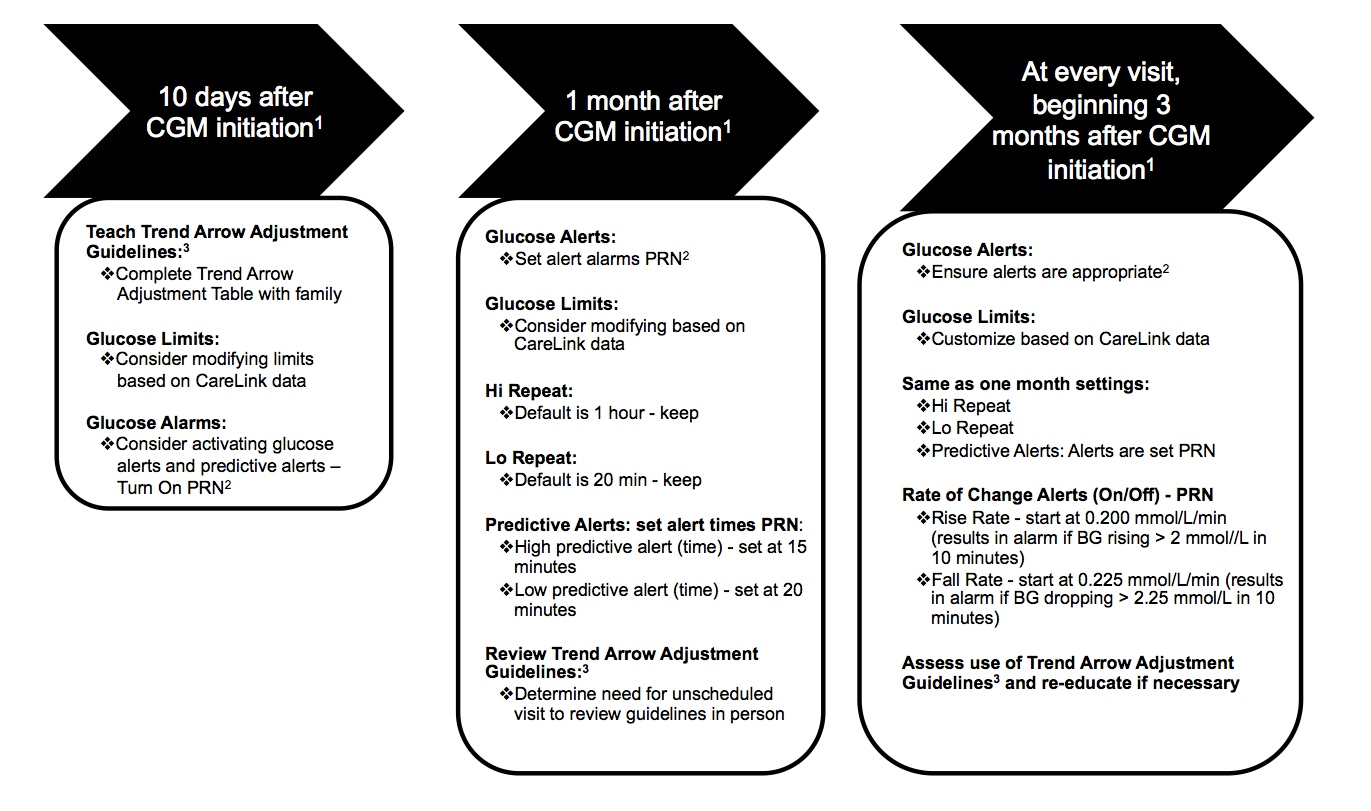

Supplement: Additional file 2: Figure S2 — Stepwise Integration of CGM Alarms. ISF = insulin sensitivity factor. 1 Delayed Group: all dates are following initiation of CGM at the 6 month visit. 2 Decision to set alarms is based on assessment of BG variability within and between days; goal is to have < 1 alarm per day. 3Study-specific guidelines developed for the CGM TIME Trial. [file 1471-2431-14-183-S2.jpeg]

### Additional File 3: Schedule of Study Visits, Telephone Contacts, and Outcome Assessments

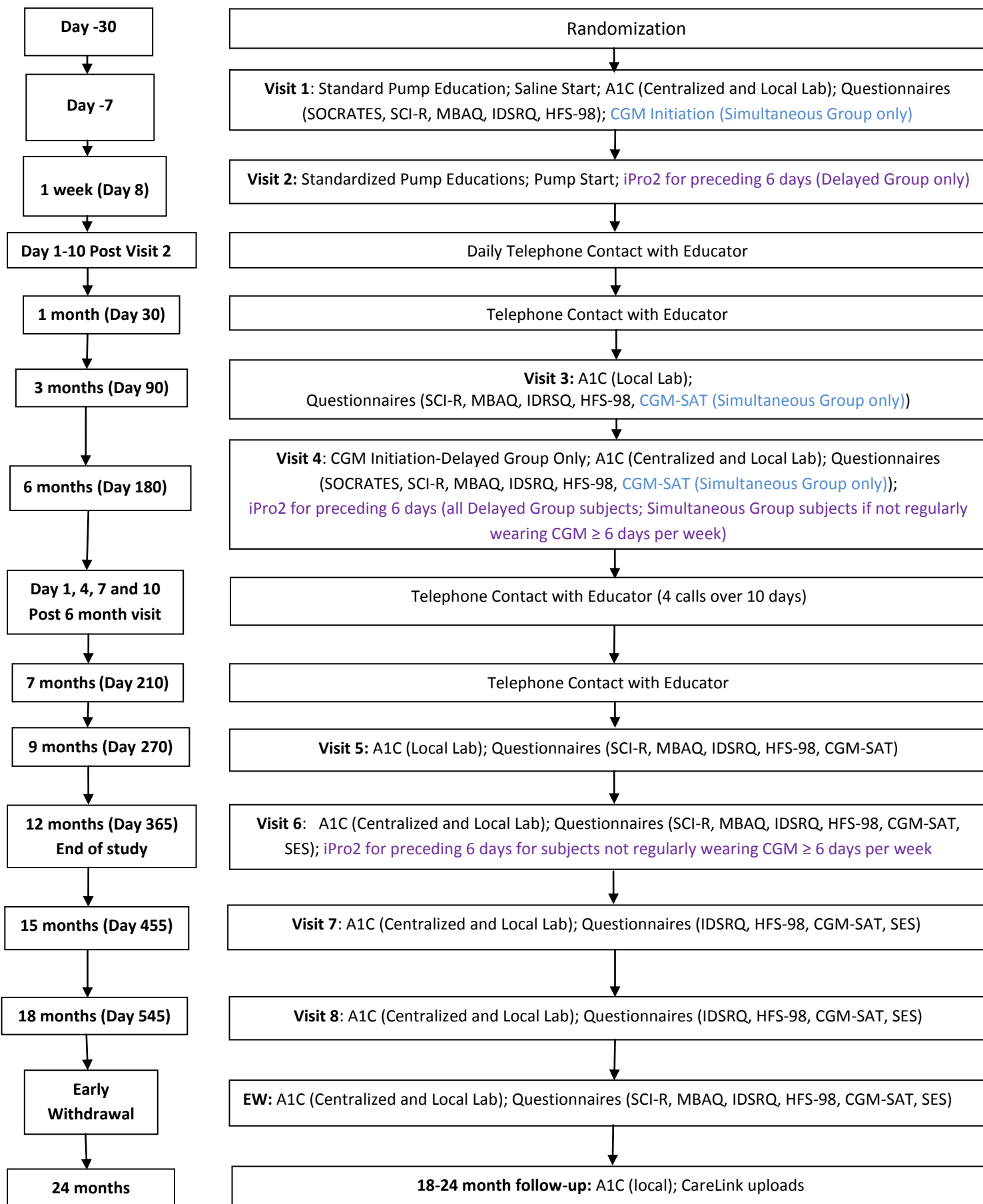

Supplement: Additional file 3: Figure S3 — Schedule of Study Visits, Telephone Contacts, and Outcome Assessments. CareLink uploads performed weekly for the duration of the trial. 24 month followup involves data from CareLink uploads and local A1Cs at 18 and 24 months. Questionnaires: SOCRATES = Stages of Change Readiness and Treatment Eagerness Scale, SCI-R = Self-Care Inventory-Revised, MBAQ = Modified Barriers to Adherence, IDRSQ = Insulin Delivery Systems Rating Questionnaire, CGM-SAT = CGM Satisfaction Scale, HFS-98 = Hypoglycemia Fear Scale, SES = Health Insurance and Socioeconomic Status Questionnaire. [file 1471-2431-14-183-S3.pdf]
